# Cleavage of Nidogen-1 by Cathepsin S Impairs Its Binding to Basement Membrane Partners

**DOI:** 10.1371/journal.pone.0043494

**Published:** 2012-08-28

**Authors:** Juliette Sage, Emmanuelle Leblanc-Noblesse, Carine Nizard, Takako Sasaki, Sylvianne Schnebert, Eric Perrier, Robin Kurfurst, Dieter Brömme, Gilles Lalmanach, Fabien Lecaille

**Affiliations:** 1 INSERM, UMR 1100, Pathologies Respiratoires: protéolyse et aérosolthérapie, Centre d’Etude des Pathologies Respiratoires, Tours, France; 2 Université François Rabelais, UMR 1100, Tours, France; 3 Louis Vuitton Moët Hennessy (LVMH-Recherche), Saint Jean de Braye, France; 4 Laboratoire de Pharmacologie Cellulaire de l’Ecole Pratique des Hautes Etudes, Centre de Recherche des Cordeliers, Paris, France; 5 Department of Experimental Medicine I, Nikolaus-Fiebiger Center for Molecular Medicine, University of Erlangen-Nuremberg, Erlangen, Germany; 6 University of British Columbia, Department of Oral Biological and Medical Sciences, Vancouver, British Columbia, Canada; University Paris Diderot-Paris 7, France

## Abstract

Cathepsin S (catS), which is expressed in normal human keratinocytes and localized close to the dermal-epidermal junction (DEJ) degrades some of major basement membrane (BM) constituents. Among them, catS readily hydrolyzed in a time and dose dependent manner human nidogen-1 (nid-1) and nidogen-2, which are key proteins in the BM structure. CatS preferentially cleaved nid-1 at both acid and neutral pH. Hydrolysis of nid-1 was hampered in murine *ctss* ^−/−^ spleen lysates pretreated with inhibitors of other classes of proteases. Nid-1 was cleaved within its G2 and G3 globular domains that are both involved in interactions with other BM components. Binding assays with soluble and immobilized ligands indicated that catS altered the formation of complexes between nid-1 and other BM components. Assuming that the cleavage of nid-1 impairs its ability to crosslink with BM partners and perturbs the viscoelastic properties of BM matrix, these data indicate that catS may participate in BM proteolysis, in addition to already identified proteases.

## Introduction

The basement membrane (BM) is a continuous sheet of specialized extracellular matrix (ECM) that regulates tissue morphogenesis and cell functions like differentiation, migration and survival by providing the scaffolding that maintains normal tissue architecture during regeneration and growth [1]. The function of the BM is closely related to its composition [2]. The main components of the BM are intertwined networks of laminins and type IV collagens, linked together by non-covalent interactions with nidogens, which also bind to perlecan, a heparan sulfate proteoglycan (HSPG). Gene knock-out studies have shown that most of the major BM components are important for the integrity of the BM [3].

Nidogens are ubiquitous BM proteins, with nidogen-1 (nid-1) being more abundant in mammals while the pattern of nidogen-2 (nid-2) distribution is distinct from nid-1. Both nidogens play a central role in the supramolecular organization of the basal laminae in tissues such as skin, muscle, lung and the nervous system [4]. Nid-1 consists of a single polypeptide chain (150 kDa) that forms three globular domains, G1 (with an apparent molecular mass of 30–35 kDa), G2 (31 kDa), and G3 (44 kDa), separated from each other by either a flexible, protease-sensitive linker (G1, G2) or a longer rigid rod-like domain (G2, G3). Though nid-1 cannot self-assemble, unlike most ECM molecules, it has several binding sites (localized to its G2 and G3 domains) for other BM components. Sequence data demonstrated that nidogens like many ECM proteins are constructed of common modules, including epidermal growth factor (EGF), thyroglobulin type-1 (Tg-1) and low density lipoprotein receptor. Similar Tg-1 domains, based on sequence motif of Cys-Trp-Cys-Val, have been found in many other proteins (saxiphilin, thyropin, testican-1) [5]. A few of them were named thyropins (thyroglobulin type-1 domain protease inhibitors) since they exhibit inhibitory activities against certain members of papain-like cysteine, aspartic proteases and metalloproteases [6]–[8]. Isolated nid-1 is readily hydrolyzed by serine proteases and metalloproteinases (MMPs) [9]–[13], however it is more resistant to degradation when complexed with laminin-111 (α1β1γ1) (the binding site for nidogens locates in gamma 1 chain) [14]. Consequently, the proteolytic degradation of nid-1 may trigger reorganization of the BM during the turnover of the extracellular matrix (ECM) that occurs in cell invasion and tissue transmigration. ECM turnover is directly correlated with the presence of proteases like MMPs, serine proteases, aspartic proteinases, and cysteine cathepsins [15]. The eleven cathepsins (B, C, F, H, K, L, S, O, V, W and X) are normally found in acidic compartments such as endosomes and lysosomes, but cysteine cathepsins also act extracellularly, particularly in acidified pericellular environment. Further, cysteine cathepsins generally exhibit limited lifetimes at neutral pH, except for catS [16]. They can hydrolyze a broad range of ECM protein, including the collagens and elastin [17]–[19], but little is known about which cysteine cathepsins are involved in the degradation of the BM in skin and how they act. CatS readily participates to the extracellular matrix turn-over because it is active and stable at neutral pH and under oxidizing conditions, has a potent elastinolytic activity and can activate other cysteine cathepsins [16], [20]. CatS is found in keratinocytes and phagocytic and antigen-presenting cells (macrophages, B lymphocytes, dendritic cells) of tissues such as the spleen, lung and skin.

In this study, immunofluorescence staining indicated that catS is localized close to the dermal-epidermal junction (DEJ) in normal skin. CatS secreted by primary cultures of human keratinocytes hydrolyzed several BM molecules, including nid-1 within a reconstituted BM. Cleavage sites were identified within G2 and G3 globular domains that are both involved in interactions with other BM components (type IV collagen, laminin, perlecan). Surface plasmon resonance and rheological analyses demonstrated that the stability of the BM and its networks were perturbed by the hydrolysis of nid-1 by catS. Thus, catS may participate with other identified proteases in the proteolysis of the skin BM.

## Materials and Methods

### Materials

Human cathepsins B, H, L, and S were purchased from Calbiochem (VWR International, Pessac, France) and catK was expressed in *Pichia pastoris* as described previously [21]. The active concentrations of these peptidases were determined by titration with L-3-carboxy-trans-2, 3-epoxy-propionyl-leucylamide-(4-guanido)-butane (E-64) (Sigma-Aldrich, St Quentin le Fallavier, France) according to [22]. Assay buffers used for cathepsins activity were either 0.1 M sodium acetate buffer, pH 5.5, 2 mM dithiothreitol (DTT) and 0.01% Brij35 (buffer A) or 0.1 M sodium phosphate buffer, pH 7.4, 2 mM DTT, 0.01% Brij35 (buffer B). Morpholinourea-leucinyl-homophenylalanine-vinyl-sulfone phenyl inhibitor (LHVS) was a kind gift from Dr. J. H. McKerrow (University of California, San Francisco, CA, USA). Laminin-211/221 (abbreviated forms corresponding respectively to chains: α2β1γ1/α2β2γ1) and type IV collagen (both from human placenta), perlecan and basement membrane extract, ECM gel (both derived from Engelbreth-Holm-Swarm (EHS) mouse sarcoma) were obtained from Sigma-Aldrich. Fibronectin (from human plasma) was from Calbiochem. Recombinant human nid-1 and nid-2 and their specific antibodies were obtained from R&D Systems (Minneapolis, USA). Recombinant mouse nid-1 and its isolated globular domains (G1, G2 and G3) were prepared as previously described [23], [24].

The antibodies used for western blot (WB) and immunofluorescence (IF) against cathepsins L and S were from R&D Systems; they were diluted to 1∶1000 for WB and 1∶50 for IF, except for catL (1∶25). Anti-catB antibodies were from Calbiochem for WB (1∶1000) and from R&D Systems for IF (1∶50). Anti-catK antibody was from Fitzgerald (Interchim, Montluçon, France) and was diluted to 1∶1000 for WB and 1∶500 for IF. Antibodies for nid-1 and nid-2 were from R&D Systems (1∶1000 for WB; 1∶200 for IF). The anti-type IV collagen antibody used for WB (1∶5000) was purchased from Abcam (Paris, France) and that for IF (1∶200) was from Novocastra (A. Menarini Diagnostics France, Rungis, France). The anti-laminin (gamma 1 chain) antibody was from Neomarkers (Thermo Fisher Scientific, Francheville, France) for WB (1∶10000) and from Novocastra for IF (clone LAM-89; 1∶200). The anti-perlecan antibody used for WB (1∶500) was from Sigma-Aldrich. Polyclonal anti-keratin antibody used for WB (1∶1000) was from Abcam.

The lack of cross reactivity of each anti-cathepsin B, L, K and S antibody was checked by western blot analysis on human cathepsins B, K, L and S (100 ng) and with keratins from human epidermis (Sigma-Aldrich) (Figure S1).

### Ethic Statement

Human abdominal skin samples were purchased from Biopredic International (Rennes, France). All samples were collected from adult patients undergoing abdominal plastic surgery and were considered as “waste” and thus were exempt from ethical approval. Helsinki principles were adhered to and participants gave written, informed consent to provide samples for research.

### Immunofluorescence

Biopsies of human skin were embedded in OCT (TissueTekSakura), frozen in liquid nitrogen and stored at −20°C. Sections (10 µm) were cut on a cryostat, placed on Superfrost+ slides (Dako, Trappes, France) and fixed in acetone at −20°C for 10 min. They were then rinsed with phosphate-buffered saline (PBS) and incubated for 30 min with PBS containing 1% BSA at room temperature. They were washed three times with PBS and incubated with the primary antibodies overnight at 4°C in a dark humid chamber. The sections were rinsed with PBS and incubated with the appropriate secondary antibody (labeled with AlexaFluor 546, 1∶200, Molecular Probes, Paisley, England) for 1 h at room temperature. Nuclei were stained with DAPI (0.1 µg/ml, Sigma-Aldrich). Negative controls were prepared without primary antibodies. The sections were given a final rinse with PBS, mounted with the Dako fluorescent mounting system, and stored at 4°C protected from light. Sections were analyzed with a SP 5 Leica confocal microscope (magnification:×630).

In parallel, cathepsins B, K, L and S were detected by immunoblot staining in whole epidermis extract from human skin (Biopredic International) by using a method adapted from [25]. Briefly, epidermis was homogenized in cold buffer containing 10 mM Hepes/KOH, pH 7.9, 10 mM KCl, 2 mM MgCl_2_, 0.1 mM DTT, 0.1% (v/v) Nonidet P40, in presence of protease inhibitors cocktail (0.5 mM Pefabloc SC, 0.5 mM EDTA, 1 mM methylmethane-thiosulfonate (MMTS), 0.04 mM pepstatin A). After centrifugation (12 000×g) during 2 min, supernatants were collected and mixed with Laemmli buffer.

### Cell Culture

Normal human adult keratinocytes isolated from abdominal skin (Biopredic International) according to [26] and immortalized non-tumorigenic keratinocyte HaCaT cells (kindly provided by Dr. N.E. Fusenig, Division of Carcinogenesis and Differentiation, German Cancer Research Center, Heidelberg, Germany, [27]) were grown to 90% confluence in keratinocyte serum-free medium (KSFM, Invitrogen, Cergy Pontoise, France) supplemented with 5 ng/mL epidermal growth factor (Invitrogen), 50 µg/mL bovine pituitary extract (Invitrogen), 100 U/mL penicillin (Invitrogen), and 100 U/mL streptomycin (Invitrogen). Cells were grown at 37°C in saturated 5% CO_2_.

### Transmigration Assays

Assays were performed in 24-well plates using a BD Biocoat growth factor reduced Matrigel chamber (BD Biosciences, Le Pont de Claix, France) with an 8-µm pore size membrane. Briefly, human keratinocytes (5×10^4^ cells per chamber) were seeded in KSFM on the upper compartment of the chamber. Supplemented KSFM was added to the lower compartment to stimulate cell migration. Cells were incubated at 37°C in saturated 5% CO_2_ for 6 h. Invasion experiments were repeated in the presence of E-64 (20 µM). Cell migration assays were performed without pretreating keratinocytes with inhibitors of other classes of proteases. The treatment with E-64 had any influence in vitro on the proliferation/survival or size of keratinocytes. Non-invading cells from the interior of the inserts were removed by using cotton-tipped swabs. Cells that migrated through the membrane and attached to the bottom of the membrane were fixed, stained with hematoxylin and counted in the whole insert using a light microscope (200×magnification). For easier comparison, the results obtained were normalized to the control condition. Values shown are the mean ± S.D.

### Western Blot Analysis and Titration of Cathepsins in Supernatants

Supernatants and cells were harvested in 0.1 M sodium acetate buffer, pH 5.5, containing protease inhibitors (0.5 mM Pefabloc SC, 0.5 mM EDTA, 1 mM MMTS, 0.04 mM pepstatin A) and stored at −80°C. The protein concentrations in supernatants and cell lysates were determined with the bicinchoninic acid assays kit (BCA protein assay kit, Interchim, Montluçon, France). The proteins in concentrated supernatants (100 µg protein/well) were separated by 15% SDS-PAGE under reducing conditions and transferred to nitrocellulose membranes. Cathepsins were detected by immunoblotting using the antibodies listed above. The cathepsins in keratinocyte supernatants were activated in 0.1 M sodium acetate buffer, pH 5.5, 5 mM DTT, 0.01% Brij35 for 3 min at 37°C. The hydrolysis of 7-amino-4-methyl coumarin (AMC)-derived fluorogenic substrate was continuously recorded at 37°C with gentle agitation, with a Gemini spectrofluorimeter (Molecular Devices, Saint Grégoire, France) with λ_exc_ = 350 nm and λ_em_ = 460 nm. Each well of the 96-well microtiter plate (Nunc) contained 20 µl supernatant in buffer A (200 µl final volume) plus the substrate. Extracellular cathepsins were titrated with E-64 (0–20 nM final) using benzyloxycarbonyl -Phe-Arg-AMC (Z-Phe-Arg-AMC) as substrate (20 µM).

### Breakdown of nid-1 from BM Matrix by Cathepsins Secreted into the Cell-free Supernatant

Five micrograms of BM matrix (ECM gel: ∼ 61% laminin, 30% type IV collagen, 7% nidogen and a trace of perlecan; Sigma-Aldrich) were incubated alone or with concentrated keratinocyte supernatants (10 µg of protein) containing protease inhibitors (0.5 mM Pefabloc SC, 0.5 mM EDTA, 1 mM MMTS, 0.04 mM pepstatin A) in buffer A or buffer B (except both contain 5 mM DTT instead 2 mM), at 37°C for 24 h. The controls were supernatants that had been incubated for 30 min with E-64 (10 µM) or LHVS (5 µM). Samples were separated by SDS-PAGE (10%) under reducing conditions followed by immunoblot staining with nid-1 polyclonal antibodies. Data from at least three independent experiments were averaged and expressed in percentage of nid-1 hydrolyzed.

### BM Matrix Degradation by Murine Spleen Lysates

Spleen lysates from catS knock-out (*ctss* ^−/−^) mice or wild-type (C57/Bl6 background) mice were kind gifts from Drs. M. Guerre-Millo and K. Clément (Inserm U872, Université Pierre et Marie Curie, Paris-6, France). Targeting of mouse catS gene and cell isolation procedures have been described in previous publication [28]. Both lysates were in 0.1 M sodium acetate buffer, pH 5.5 plus a cocktail of peptidase inhibitors (1 mM PMSF, 1 mM EDTA, 0.04 mM pepstatin A, 1 mM MMTS). Their protein concentrations were determined by BCA assays. Cell lysates (10 µg of proteins) were incubated with ECM gel (10 µg) in buffer B (pH 7.4) at 37°C for 18 h. Reaction products were reduced and separated by SDS-PAGE (10%) and the gels were transferred to nitrocellulose membranes for nid-1 immunodetection.

### Rheology of the BM Matrix

The viscosity and elasticity of samples of ECM gel (8 mg/ml) alone or ECM that had been incubated with catS (60 nM) in buffer A (pH 5.5) for 36 h at 25°C were measured using a rotational rheometer (ARES, RFS III, TA Instruments, France) according to [29]. The control was ECM that had been incubated with catS inactivated by E-64 (10 µM) samples. The rheometer was equipped with a horizontal plate bearing a cone. Samples (600 µl) were placed between the upper cone and the plate which was heated to 37°C to allow gel formation. Shear experiments were performed on the sample up to 1% strain, with an angular velocity that oscillated sinusoidally. Both viscosity (G’) and elasticity (G”) parameters were measured in the linear viscoelastic regimen as a function of the frequency of the angular velocity from 0.1–1 Hz. Data were analysed with TA Orchestrator v 7.2.0.2 software. The integrity of BM components (type IV collagen, laminin, perlecan and nid-1) in the different samples was controlled by immunoblot staining.

### Hydrolysis of Nid-1 and Nid-2 by CatS

Samples of nid-1 (143 nM corresponding to 50 ng protein) were incubated with cathepsins B, H, K, L and S (1 nM) in buffer A (pH 5.5) for 0–120 min at 37°C. The experiment was repeated in buffer B (pH 7.4) for 30 min. Samples were separated by SDS-PAGE (10%), and transferred to nitrocellulose membranes for nid-1 immunodetection. They were incubated with polyclonal goat anti-nidogen-1 (1∶1000, in PBS 0.1% Tween 20, 5% skim milk powder) overnight at 4°C, washed three times with PBS, and incubated with peroxidase-conjugated anti-goat IgG (Sigma-Aldrich, 1∶5000, in PBS 0.1% Tween 20, 5% skim milk powder) for 1 h at room temperature. Nid-1 was visualized using the enhanced chemiluminescence method (Amersham Biosciences, France) according to the manufacturer’s instructions and signal densities were measured using the NIH image software (version 1.6.3). The densitometric measurements from three independent experiments were averaged and expressed as the percentage of nid-1 remaining.

Cleavage sites were identified by N-terminal sequencing (Laurent Coquet, Plateforme de Protéomique de l’IFRMP23, Université de Rouen, France). The sequence of human recombinant nid-1 starts with a human CD33 signal peptide (Met^-25^-Ala^−10^), a poly-histidine sequence (9×His) and a L^1^SRQE sequence in which Leu corresponds to the first residue of human nid-1.

Mouse nid-1 (50 ng) and its isolated globular domains; G1, G2 and G3 (100 ng) were incubated with catS (10–50 nM) in buffer A at 37°C and aliquots were removed at intervals from 10 to 240 min. Proteolysis products were separated by SDS-PAGE (10% for nid-1; 12% for isolated domains) and detected with the polyclonal anti-nid-1 antibody.

Human nid-2 (1.4 µM) was incubated with cathepsins B, K, L and S (10 nM) in buffer A for 5–60 min at 37°C, and with catS (4 nM) for 0–30 min at 37°C in buffer B; catS inactivated with E-64 was used as control. Proteins were separated by SDS-PAGE (10%) under reducing conditions and transferred to nitrocellulose membranes. Nid-2 was immunodetected by western blot, using a goat-anti-nid-2 antibody (1∶1000) and an anti-goat IgG-peroxidase conjugate (1∶5000) and quantified as described above.

### Hydrolysis of isolated basement membrane constituents and reconstituted BM matrix by cathepsins B, K, L and S

Cathepsins B, K, L and S (10–100 nM) were incubated with laminin-211/221 (3.5 µg), type IV collagen (12 µg), perlecan (3 µg), and fibronectin (5 µg) for 4 h at 37°C in buffer A. Cathepsins that had been incubated with the specific cysteine protease inhibitor E-64 (1 µM) were used as controls. Cathepsins B, K, L and S (10–200 nM) were incubated for 4 h at 37°C with 60 µg ECM gel in buffer A. As controls, cathepsins preincubated with E-64 (1 µM) for 30 minutes were used. Reactions were stopped by adding Laemmli sample buffer and proteins were separated by SDS-polyacrylamide electrophoresis on 10% gels. The gels were stained with Coomassie blue.

### Enzyme-Linked Immunosorbent Assays

Laminin-211/221, type IV collagen and perlecan (500 ng) in 50 mM sodium carbonate-bicarbonate buffer, pH 9.6 were immobilized on the surface of plastic microtiter wells (Nunc, VWR, International, Pessac, France) by incubation overnight at 4°C. Non-specific binding sites were blocked with PBS containing 2% BSA (Sigma-Aldrich). Nid-1 (0–250 ng) prepared in buffer A (pH 5.5) was placed in each well and the plate was incubated for 3 h at room temperature. The wells were then incubated with goat anti-nid-1 antibody (1∶500), followed by anti-goat IgG-peroxidase conjugate (1∶10000), then 0.04% (v/v) H_2_O_2_ and orthophenylene diamine (0.4 mg/ml) in substrate buffer (50 mM phosphate, 20 mM citric acid, pH 5.5). The reaction was stopped with 3 M H_2_SO_4_. Absorbance was measured at 492 nm in a plate reader (VersaMax, Molecular Devices, St Grégoire, France). Similar experiments were performed with fragments of nid-1. Briefly, nid-1 (25 ng) was incubated with catS (5 nM) in buffer A or buffer B for 5–60 min and hydrolysis was stopped by adding E-64 (100 nM). Samples of the reaction mixture were then placed in the wells of 96-well plates that were coated with ligand (laminin-211/221, type IV collagen and perlecan) and interactions were monitored as for intact nid-1.

### Surface Plasmon Resonance (SPR) Assays

The binding of nid-1 to laminin-211/221, type IV collagen and perlecan was measured by SPR using the BIAcore™ T100 system (BIAcore, GE Healthcare Europe GmbH, France). Nid-1 (20 µg/mL) was immobilized on the CM5 sensor chip by the carbodiimide coupling procedure at a flow-rate of 5 µl/min. The surface of the carboxymethylated sensor chip was activated by adding 0.2 M 1-ethyl-3-(3-dimethylaminopropyl)-carbodiimidehydrochloride) and 0.05 M N-hydroxysuccinimide. The nid-1 coupled to the activated chip in 10 mM sodium acetate pH 4.0 provided 1500 resonance units (RU). Residual activated esters were blocked with 1 M ethanolamine pH 8.5. For controls, one surface was activated and blocked under identical conditions without coupling nid-1. The binding of nid-1 to various ligands (laminin-211/221, 25 µg/mL; type IV collagen, 80 µg/mL and perlecan, 10 µg/mL) was studied in 10 mM Hepes, 150 mM NaCl, pH 7.4, with 0.05% Tween 20 at a flow-rate of 10 µl/min for 3 min. CatS (0–50 nM), or cathepsins B, K, L (10 nM) in 10 mM Hepes, 150 mM NaCl, pH 7.4, with 0.05% Tween 20 containing 2 mM DTT were injected at the same flow-rate for 3 min, followed by a dissociation phase of 3 min. The controls were buffer or heat-inactivated catS, injected as the second ligand. Bound ligands were removed from the immobilized protein with 0.01 M NaOH and 1 M NaCl was used to regenerate the chip.

### Statistical Analysis

Statistical differences between two groups were calculated using Student’s one-way t-test followed for keratinocytes migration, nid-1 and nid-2 breakdown and for ligand binding assays. Data from western blot analysis of nid-1 and nid-2 and BM components are expressed as means ± S.D of three separated experiments. A *P* value of less than 0.05 was considered statistically significant (*, *P*<0.05; **, *P*<0.01).

## Results

### Expression and Involvement of Cathepsins B, K, L, S in the Migration of Keratinocytes through a Reconstituted BM

**Figure 1:**
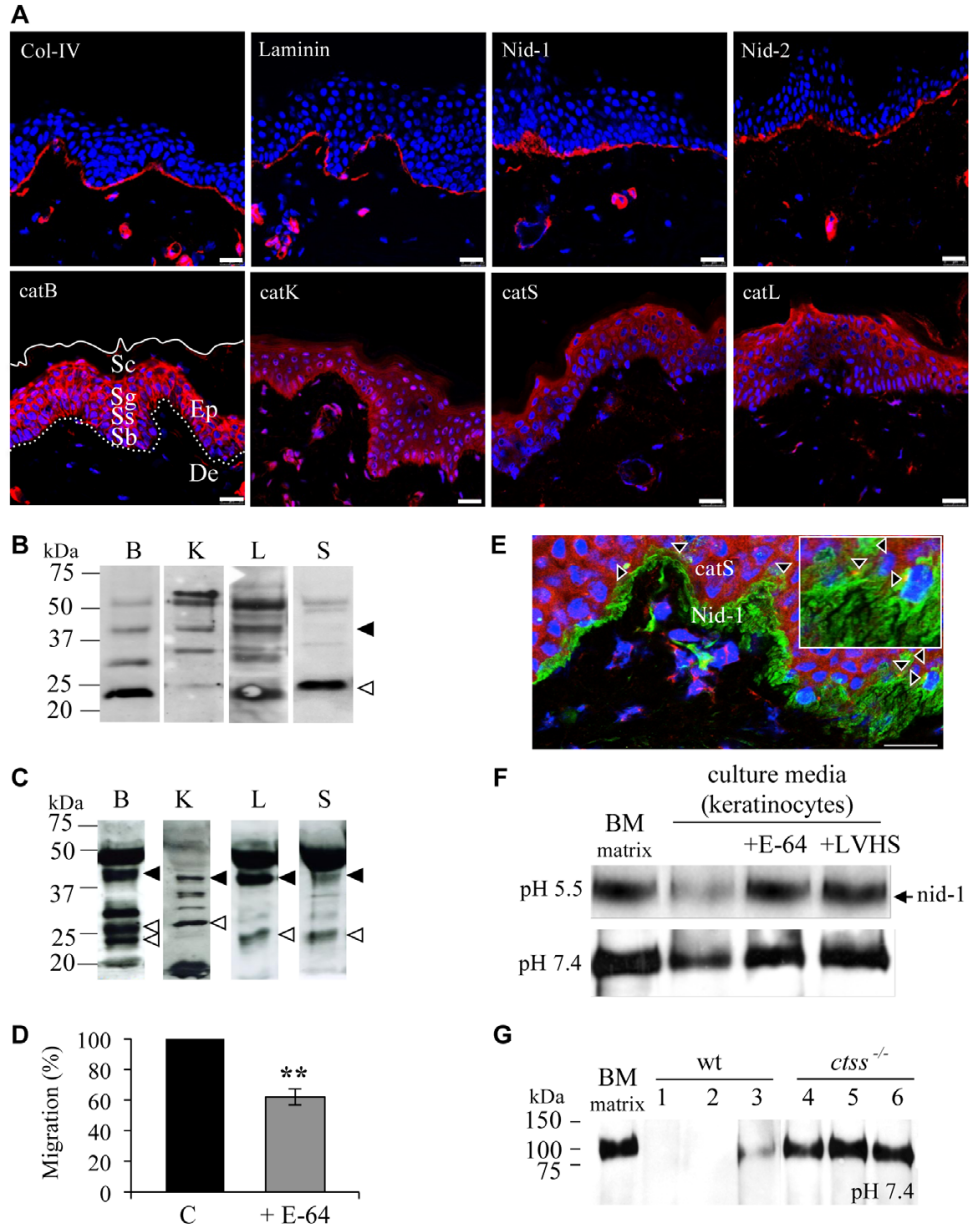
Major BM components and cysteine cathepsins B, K, L, S in the epidermis of human skin. (A) Immunofluorescence labeling of major BM components (type IV collagen, laminin, nid-1 and -2) and cathepsins B, K, L, S. The nuclei of keratinocytes were stained with DAPI. Ep: epidermis; De: dermis, Sc: stratum corneum, Sg: stratum granulosum, Ss: stratum spinosum, Sb: stratum basale. The continuous line indicates the outermost layer of the skin, the dashed line indicates the basal lamina. Bars correspond to 25 µm. (B) Expression of pro- and mature cathepsins B, K, L and S in human skin epidermis. The dark arrow indicates the proform of cathepsin and the light arrow indicated the mature form. Intermediate bands correspond to uncompleted maturation. (C) Samples of conditioned culture medium of human primary keratinocytes were concentrated (x 200), separated (100 µg protein) by SDS-PAGE (15%) under reducing conditions. (D) Transwell BM matrix migration assays (n = 5 separate experiments) by keratinocytes (C: control) treated with E-64 (**, *P*<0.01 when compared to control). (E) Double immunofluorescent labeling of catS and nid-1 in human skin and confocal laser microscopy analysis. Secondary antibodies were Alexa Fluor 546 anti-goat (catS: red) and Alexa Fluor 488 anti-mouse (nidogen-1: green). Magnification (insert) of the lower panel of the image (x 3). Arrowheads indicate cells containing catS and nid-1 close together (yellow). Scale bar = 25 µm. (F) BM matrix (5 µg) was incubated alone or with concentrated keratinocyte supernatant (10 µg protein) at pH 5.5 or 7.4 for 24 h. For controls, supernatants were pre-incubated for 30 min with E-64 or LHVS. Data represent immunoblot analysis with nid-1 antibodies from at least 3 independent experiments. (G) Pretreated lysates (10 µg) of wild-type C57/Bl6 mouse spleen (wt, lysates: 1–3) and catS-deficient spleen (*ctss^−/−^*, lysates: 4–6) were incubated (pH 7.4, 37°C) for 1 h in order to inactivate all cysteine cathepsins, except catS. BM matrix (10 µg) was then incubated alone or with cell lysates (pH 7.4, 37°C) for 18 h. Immunoreactive nid-1 was revealed by western blot. Assays were performed on different samples (n = 15), which yielded similar results and a representative of two independent experiments is presented.

The dermal-epidermal junction (DEJ) zone of biopsies of European descent healthy skin (Fig. 1A) contained type IV collagen, laminins (the major isoforms in skin BM are laminin-332 and laminin-511), nid-1, and nid-2 in a typical linear-interrupted pattern. The dermal blood vessels also contained these proteins, but they were not detected in keratinocytes (nuclear DAPI staining). CatB was detected in keratinocytes throughout the epidermal layers, as reported by Brix et al. [30]. The stratum basale, spinosum and granulosum contained intense catB immunoreactivity, but the stratum corneum was only faintly stained. CatB was found in the vesicles of the perinuclear region of proliferative keratinocytes (stratum basale), while catB was more broadly distributed throughout the upper layers (stratum spinosum and granulosum). The pattern of catK staining in the epidermis showed intense staining in the stratum corneum and stratum basale. CatL was found in keratinocytes in all epidermal layers except the stratum corneum. Like catB, catS was detected in all layers of the epidermis, particularly in the stratum spinosum and stratum basale. CatS was mainly detected in keratinocytes and in cellular protrusions between keratinocytes, with prolonged extensions close to the BM (Fig. 1A). In parallel, both pro- and mature forms of cathepsins B, K, L and S were detected by immunoblot staining in whole epidermis extract from human skin (Fig. 1B). Intermediate bands correspond to uncompleted maturation. We next identified the extracellular cathepsins produced by human keratinocytes, prior to evaluating their role in BM hydrolysis. Similarly, their concentrated cell-free supernatants revealed the presence of both zymogens and mature forms of cathepsins B, K, L and S (Fig. 1C). The presence of pro-cathepsins in the culture medium supports the concept that keratinocytes secrete cathepsins that can be autocatalytically activated [31]. Similar results were obtained with the immortalized HaCaT cells (data not shown). The total cysteine cathepsin activities in the conditioned media of cultures of primary keratinocytes (112±1 nmol/g protein) and HaCaT cells (79±1 nmol/g protein) were similar and accounted for ∼10% of the active concentrations measured in whole cell lysates.

The relative contribution of cathepsins in BM hydrolysis was investigated using a cell migration assay through a reconstituted BM matrix, (mimicking the natural BM secreted by adherent cells), using the broad spectrum cysteine cathepsins inhibitor E-64. Inhibition of cysteine cathepsins by E-64 impaired significantly keratinocytes migration (38±5%, *P*<0.01) through transwell chambers coated with BM matrix (Fig. 1D). Similar results were obtained with the membrane permeable E-64d (data not shown). This strongly suggests that human cysteine cathepsins mediate BM matrix degradation and promote with other identified proteases cell migration. Double immunofluorescence staining indicated that the distribution of catS is adjacent to nid-1 throughout the BM and its appendages in human skin biopsies (Fig. 1E). Higher magnifications showed that the staining of catS partly overlapped with that of nid-1, suggesting that the enzyme interacted with nid-1 in the vicinity of the BM. We then analyzed the contribution of catS secreted by human keratinocytes to the cleavage of nid-1 in BM matrix. Since catS is the only cathepsin that remains active after prolonged exposure to neutral pH, assays were performed at both pH (5.5 and 7.4). BM matrix incubated for 24 h with concentrated conditioned culture medium at pH 5.5 had lost almost all their nid-1 (90±0.5%, p<0.01) (Fig. 1F) and two thirds of their nid-1 (69±6.6%, p<0.01) when incubated at pH 7.4. Supernatants whose cathepsins had been irreversibly inactivated with morpholinourea-Leu-Hph-vinyl-sulfone (LHVS) or E-64 did not hydrolyze nid-1. The proteolysis of nid-1 by extracellular cathepsins at both acidic and neutral pH was confirmed with HaCaT cells as well (data not shown). However, nid-1 was not hydrolyzed by pretreated spleen lysates from catS-deficient mice (*ctss* ^−/−^) at pH 7.4 confirming the specific involvement of catS among the cathepsins tested (Fig. 1G). Western blot of BM matrix with an anti-nidogen-1 antibody showed that the ∼90 kDa band is the major form of nid-1 in the commercial BM matrix extract (ECM gel, Sigma-Aldrich). It has to be noticed that the 150 kDa form of unprocessed nid-1 is known to be highly susceptible to endogeneous proteolysis (particularly within its N-terminal domain G1) during pre-extraction tissue and that N-terminally cleaved form of ∼90 kDa nid-1 was already described in other murine BM tissues extracts. However, G2 and G3 domains are conserved and still bind to BM partners (type IV collagen, laminins, perlecan, fibulins) [11]. Experiments were also performed with an isolated nidogen/laminin complex from EHS. Western blots showed that catS cleaved readily higher forms of nid-1 (∼120 kDa, ∼150 kDa and ∼195 kDa) after 1 h incubation at pH 5.5 (data not shown). In addition, catS degraded efficiently, in a dose-dependent manner soluble preparations of major BM constituents: laminin-211/221, type IV collagen, perlecan, fibronectin and the BM matrix extract (Fig. S2A, B). All these results support that catS may participate with other proteases in the hydrolysis of major components of BM.

### Alteration of BM Matrix Viscoelastic Properties by CatS

**Figure 2:**
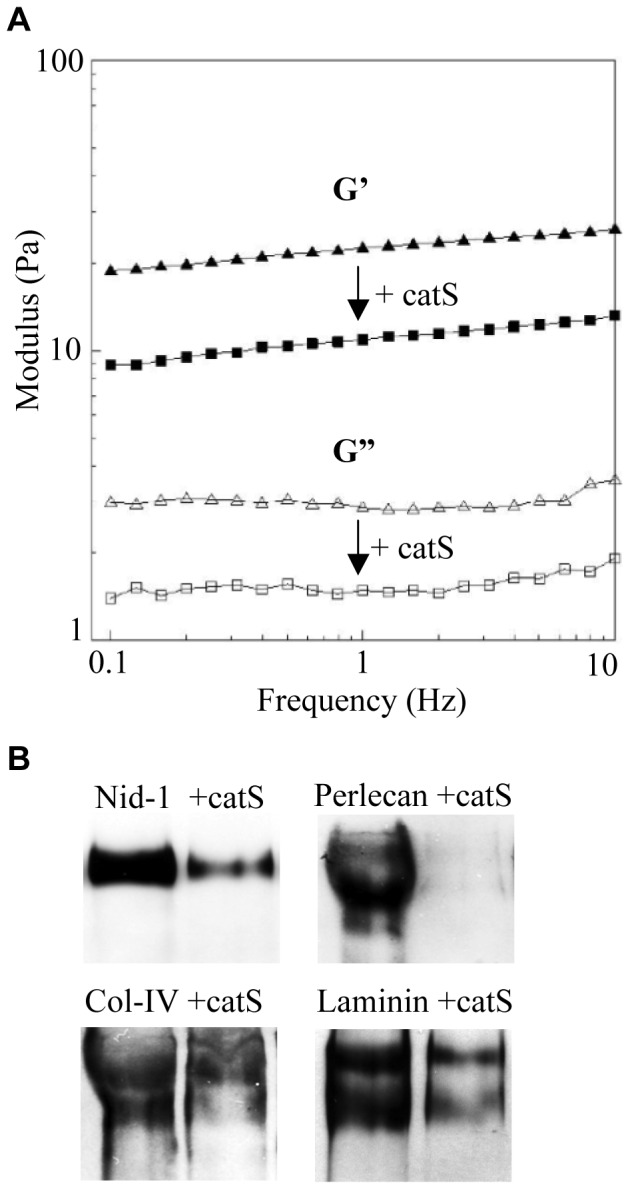
Influence of catS on the visco-elastic properties of BM matrix. (A) G’ (elasticity: black symbol) and G” (viscosity: white symbol) moduli of BM matrix (ECM gel: 8 mg/ml) without (C: control, triangles), or with catS (60 nM, squares) were determined by rheometry. A representative of three independent experiments is shown. (B) Western blot analysis of major BM constituents of ECM gel (nid-1, perlecan, laminin and collagen IV) with or without catS.

We investigated the influence of catS on the mechanical properties (elasticity and viscosity) of the BM extract by rheological analysis (Fig. 2A). Matrix (ECM gel alone or incubated with catS) was subjected to small amplitude oscillatory shearing and G’ (elasticity) and G” (viscosity) moduli were recorded as a function of the frequency. Gelified ECM gel (8 mg/ml) had a typical solid material-like profile; the G’ modulus was greater than the G” modulus throughout the frequency range, and were similar to the values for Matrigel (another commercial source of BM extract from EHS), at the same concentration [29]. CatS reduced both moduli (elasticity and viscosity) by one-half, but heat-inactivated catS did not alter either property (data not shown). Immunoblot analysis confirmed that these alterations in the visco-elastic properties of the BM were due to the hydrolysis of nid-1 as well as other major constituents including type IV collagen, laminins, and perlecan (Fig. 2B).

### Identification of Cleavage Sites of Recombinant Nid-1 by CatS

**Figure 3:**
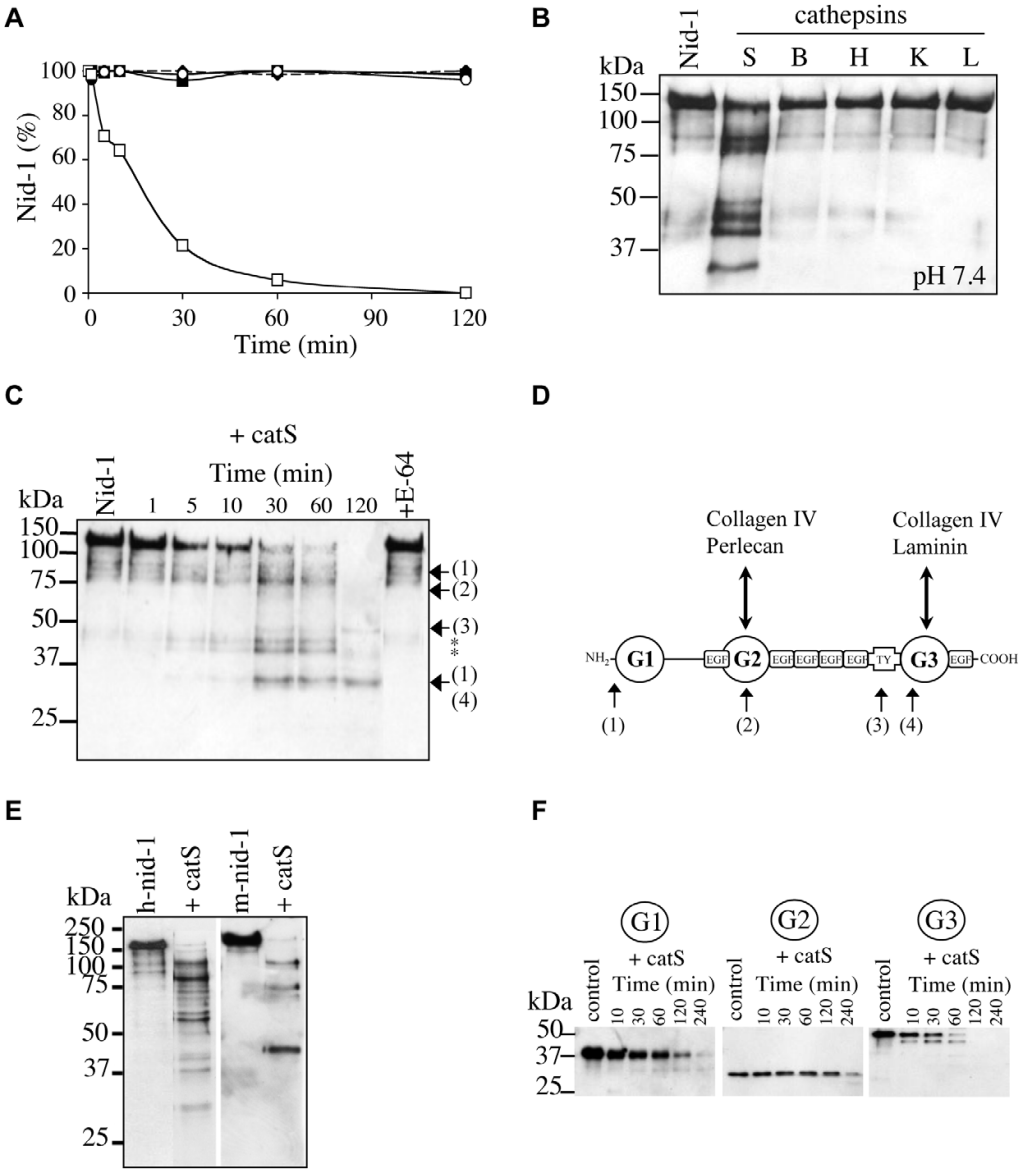
Proteolysis of recombinant nid-1 by catS and location of the cleavage sites. (A) Hydrolysis (0–120 min) of human nid-1 by cathepsins B (•), L (○), K (♦), H (▪) and S (□). Nid-1 was incubated with each cathepsin at pH 5.5 (enzyme:substrate ratio = 1∶143, w/w). Residual nid-1 was quantified by densitometry. (B) Hydrolysis of human nid-1 by cathepsins B, H, K, L and S at pH 7.4 for 30 min (enzyme:substrate ratio = 1∶143, w/w). (C) Human nid-1 (50 ng) was incubated with catS (1 nM) at pH 5.5. Fragments were identified by N-terminal sequencing and their positions in the nid-1 sequence (1–4) are indicated. Asterisks indicate N-terminal fragments for which no sequence was obtained. (D) Diagram showing the nid-1 domains and location of protein binding sites. CatS cleavage sites are indicated as: (1) corresponds to the N-terminal sequence ^-25^MPLLLL of rec. human nid-1; (2) corresponds to ^460^APVGGI; (3) corresponds to ^694^HXLGAA and (4) corresponds to ^860^XXPEGI where X stands for unidentified residues. (E) Hydrolysis of human and mouse nid-1 (50 ng) by human catS (10 nM) at pH 5.5; incubation for 10 min. (F) Recombinant mouse G1 domain (100 ng) was incubated with catS (50 nM) at pH 5.5 for 10, 30, 60, 120 and 240 min. Similar assays were performed with mouse G2 and G3 domains. Anti-nid-1 antibody was used for western blot analysis.

CatS (1 nM) cleaved 50% of nid-1 within 15 min and to 100% in 2 h resulting in one major fragment of ∼34 kDa and a minor of ∼49 kDa (Figs. 3A, 3C). Cathepsins B, L and K were less efficient (enzyme:substrate ratio = 1∶143, w/w); undigested nid-1 still remained after incubation for 2 h (Fig. 3A). CatL cleaved ∼35% of nid-1 in 4 h (data not shown). Unlike other cathepsins, catS also hydrolyzed nid-1 at neutral pH (40% of nid-1 was cleaved after 30 min and 75% cleaved after 120 min) (Fig. 3B). Sequencing the N-terminals of hydrolysis products identified the main cleavage sites (→) of nid-1 as LLPL^459^↓APVG (within the central part of the G2 domain), HERE^693^↓HILG (in the thyroglobulin-like domain) and RQDL^859^↓GSPE (at the N-terminal part of the G3 domain) (Fig. 3D). Some bands (*) gave no interpretable results (Fig. 3C). Western blot analyses of the catS mediated hydrolysis of recombinant human and mouse nid-1 (Fig. 3E), which is more resistant to proteolysis [11], revealed significant differences. CatS digests of mouse nid-1 contained only four major bands while numerous fragments were detected in the human nid-1. We also digested the recombinant globular domains (G1 to G3) of mouse nid-1 with catS (Fig. 3F). The mouse G2 domain appeared to be the most resistant to proteolysis, probably due to the presence of a N-glycosylation site that is absent in human nid-1 [32]. The G1 and G3 domains were completely hydrolyzed after incubation with catS for 4 h, but approximately 30% of the G2 domain remained intact at this time.

### Hydrolysis of Recombinant Nid-2 by CatS

**Figure 4:**
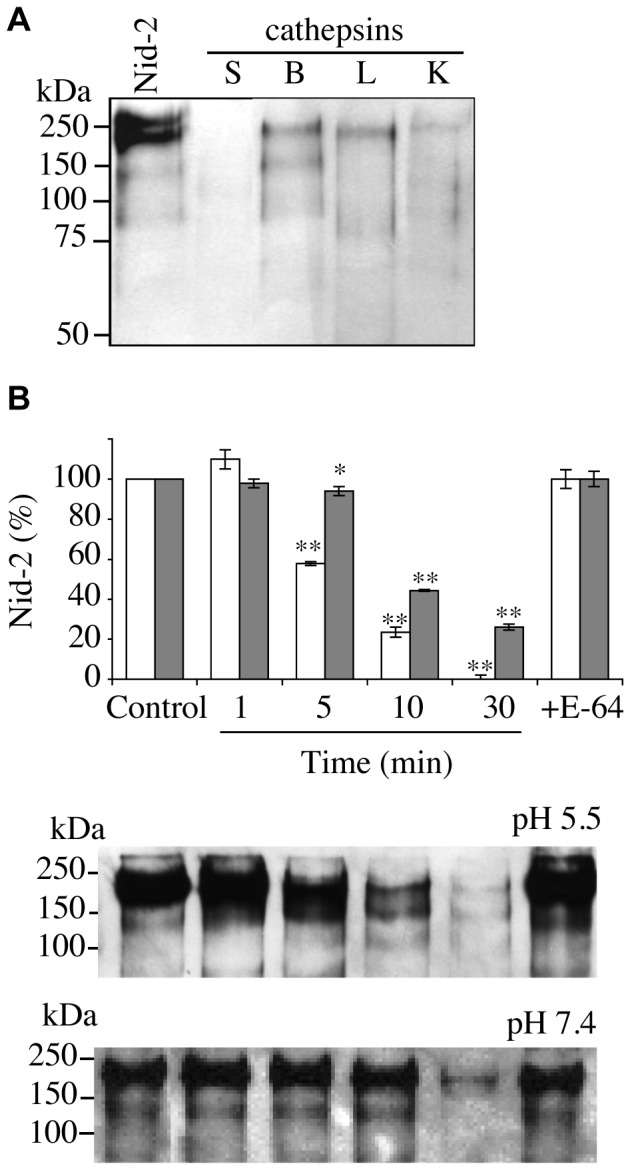
Hydrolysis of nid-2 by catS. (A) Western-blot of human nid-2 incubated with catS, catB, catL and catK at pH 5.5 for 30 min (enzyme:substrate ratio = 1∶143, w/w). (B) Densitometry analysis of nid-2 after incubation with catS (1, 5, 10, 30 min) at pH 5.5 (white bar) and pH 7.4 (grey bar). E-64 inactivated catS was used as a control. The percentage of residual nid-2 was expressed as means ± S.D (*, *P*<0.05 and **, *P*<0.01 when compared to control). A representative of three independent western blots at both pH is shown.

Nid-2 (200 kDa) is longer than nid-1, and there are significant differences in their link and rod domains. Its sequence is 46% identical to that of nid-1. The two nidogen isoforms have similar structures and the same modular structure of three globular domains (G1–G3) [33]. We therefore evaluated the sensitivity of human nid-2 to proteolysis by catS (Fig. 4). CatS was by far the most efficient cathepsin tested at cleaving nid-2 after 30 min incubation, at pH 5.5 (Fig. 4A). Like nid-1, nid-2 was readily cleaved by catS at pH 5.5, while 30% of nid-2 remained uncleaved at neutral pH (Fig. 4B). Our results therefore show that both nidogen isoforms are susceptible to proteolysis by catS.

### CatS and the Binding Properties of Human Nid-1

**Figure 5:**
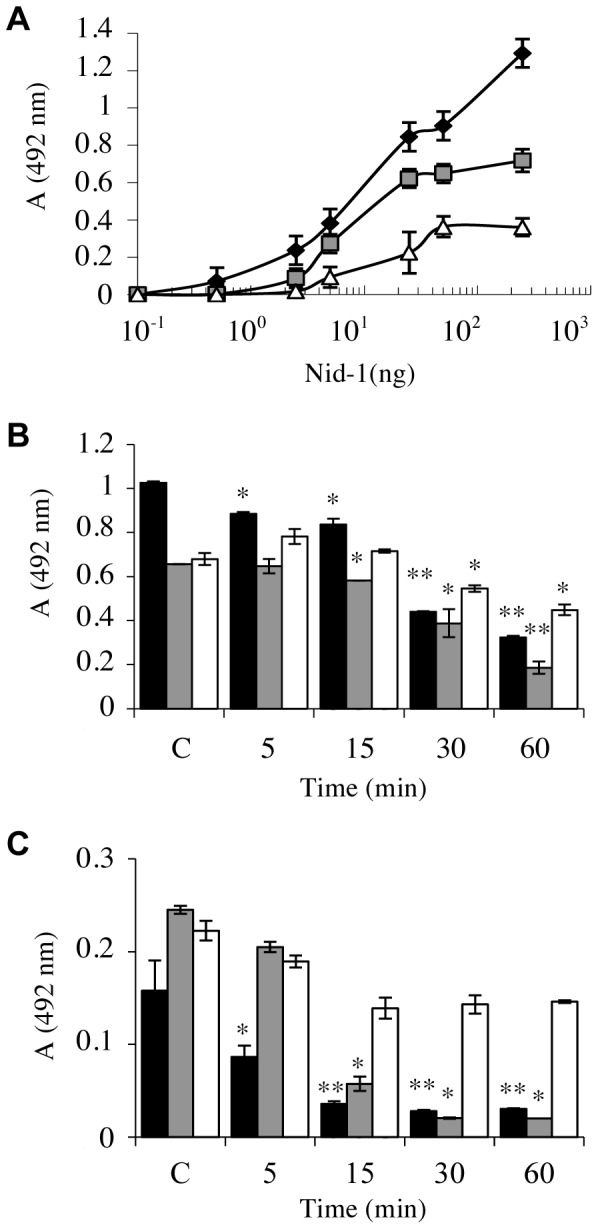
Influence of catS on nid-1 binding to BM constituents. (A) ELISA results showing the binding at pH 5.5 of undigested nid-1 (0–250 ng) to immobilized ligands (500 ng): laminin-211/221 (black diamond), collagen IV (dark grey square) and perlecan (white triangle). (B) ELISA results showing the binding at pH 5.5 of nid-1 (25 ng) pre-incubated with catS (5 nM) for 5–60 min to immobilized ligands (500 ng): laminin-211/221 (black bar), collagen IV (dark grey bar) and perlecan (white bar). Controls were performed using undigested nid-1 (25 ng) at pH 5.5. (C) The same experiment was repeated at pH 7.4. Experiments were performed three independent times (*, *P*<0.05 and **, *P*<0.01 when compared to control).

We analysed the binding of soluble nid-1 to laminin-211/221, type IV collagen and perlecan using an enzyme-linked immunosorbant assay with anti-nidogen-1 (Fig. 5). Nid-1 was bound to each of the immobilized ligands in a concentration dependent manner (Fig. 5A). The dose-response profiles were all similar, despite its apparently higher affinity for laminin-211/221. The half-maximal reactions occurred around 1–2 nM nid-1, in agreement with the apparent dissociation constant, Kd [34]. Previous studies showed that human nid-1 had weaker affinities for collagen IV and perlecan than for laminin [11]. We next examined the binding of a mixture of nid-1 fragments produced by incubation with catS (Fig. 3C) to immobilized ligands. The nid-1 bound to laminin-211/221 and type IV collagen had decreased >3-fold after incubation for 60 min (Fig. 5B) but the binding to perlecan had decreased only 1.6-fold, probably due to its contamination with laminin during the extraction procedure, as described elsewhere (1–2.5%) [35]. The binding patterns at pH 7.4, were similar, although the interactions between nid-1 and its ligands were weaker. Despite an identical molarity of both buffers, the ionic strength of sodium phosphate buffer (0.265 M) is different to that observed at pH 5.5 (0.087 M) (theoritical values were obtained by using a free software developed by Rob Beynon: www.liv.ac.uk/buffers/buffercalc.html) (Fig. 5C). A such difference may therefore affect nidogen-1 binding to BM partners. Thus, the hydrolysis of nid-1 by catS generates fragments that bind to immobilized laminin-211/221, collagen IV and perlecan significantly more weakly than does intact nid-1.

### Interactions of Nid-1 with its BM Partners in the Presence of CatS

**Figure 6:**
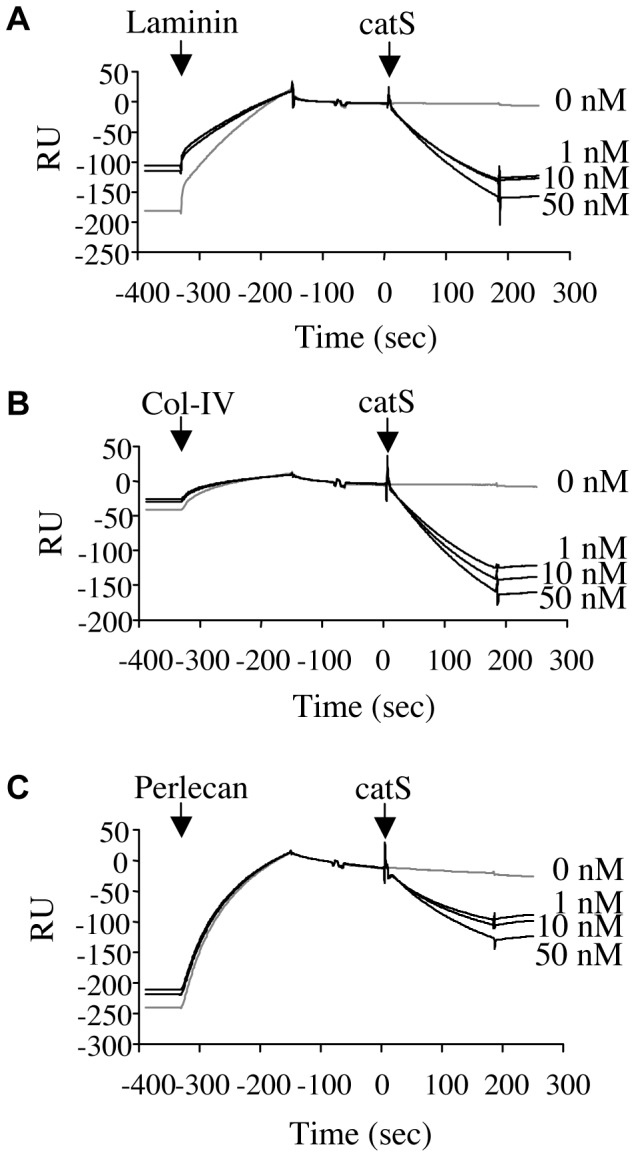
Dynamic analysis of catS activity on nid-1 complexes by surface plasmon resonance. Sensorgrams of SPR analysis show the binding of intact laminin-211/221 (A), collagen IV (B) or perlecan (C) to immobilized nid-1. Stable complexes were incubated with catS (1–50 nM) added at t = 0 sec.

Surface plasmon resonance analysis of the binding of immobilized nid-1 to laminin-211/221, type IV collagen, and perlecan at pH 7.4 gave typical association curves (Fig. 6). Nid-1 bound type IV collagen less strongly than either laminin-211/221 or perlecan. Addition of catS immediately decreased the resonance unit (RU) in a dose-dependent manner (a 50% decrease within 1 min). CatS completely dissociated at pH 7.4 the nid-1-laminin-211/221 and nid-1-type IV collagen complexes, in agreement with the ligand binding assays but not the nid-1-perlecan complexes. No decrease in RU signal was measured in presence of buffer or heat-inactivated catS. There was a small increase in the RU signal when heat-inactivated catS was added to complexes of nid-1 and laminin-211/221, collagen IV or perlecan (data not shown). This suggests that catS may interact with BM proteins, as do cathepsins B and L [36], [37]. Cathepsins B, L and K did not modify the binding of nid-1 to the other BM proteins at pH 7.4 (data not shown).

## Discussion

While numerous studies have shown that catS takes part in intracellular and extracellular processes, most of its specific biological substrates have not been completely characterized [16]. The high elastinolytic and collagenolytic activities of catS and its proteolytic activity at neutral pH indicate that catS can disrupt BM assembly [20], [38]. Despite the pH gradient from the stratum basale (pH 7.4) of the epidermis to the outer stratum corneum (pH 4.5) [39], cells may produce vacuolar-type H^+^-ATPase to acidify the pericellular space, so triggering cysteine cathepsin activity [31]. Immunofluorescence studies showing catS-positive vesicles in the epidermis, particularly in the stratum basale, suggest that catS contributes to the *in situ* degradation of BM constituents and particularly nid-1, a key protein with perlecan in the formation, organization, and stabilization of the epidermal BM [40].

We have shown that catS hydrolyzes readily major BM components: type IV collagen, laminin-211/221, perlecan, fibronectin, and particularly nid-1 and nid-2, more efficiently than all other cysteine cathepsins tested. The fact that catS readily hydrolyzes both nidogen isoforms adds support for its role in BM degradation in specific organs. We have also identified new critical sites in the G2 and G3 domains of human nid-1 whose cleavage abolishes the capacity of nid-1 to bind to other BM proteins. These two domains are essential for the formation of ternary complexes between major BM components [4]. The major one encompasses the first epidermal growth factor (EGF) repeat, which contains an RGD sequence that interacts with many integrin receptors, in the connecting rod-like domain. The second RGD independent attachment site lies in the cysteine–rich EGF repeat of the G2 domain. The proteolytic cleavage in the thyroglobulin type-1 region (Tg-1) of the nid-1 rod domain is rather surprising, since some Tg-1 domains (equistatin, testican-1), despite their amino acid sequence similarity, can inhibit several classes of proteinases, including cysteine cathepsins and aspartic cathepsin D [8], [41]. However, certain Tg-1 domains can also be good substrates for some cathepsins, due to steric hindrance between the Tg-1 domain and the active site of the enzyme [42]. In addition, the testican-1 Tg-1 domain exhibits both features with its target cysteine cathepsins [43]. Indeed, among proteases tested only cathepsins K and L cleaved the Tg-1 domain of testican-1. An important characteristic of Tg-1 domain of testican-1 is its selectivity in inhibiting only catL while other enzymes tested were not affected (papain, cathepsins B, K, S, X, cathepsin D and trypsin). Further, entactin (also known as nidogen) was shown to exhibit no apparent inhibitory activity against papain [44]. The widely distributed Tg-1 domains may have other features than peptidase inhibition, like the Tg-1 domains of the insulin-like growth factor binding protein-6 (IGFBP-6) which were shown to be involved in the binding of IGFs [45]. Thus, the physiological function of the Tg-1 motif of nidogen-1 towards proteases remains to be explored. Impairment in the nid-1 site (G3 domain) that binds laminin inhibits BM formation in human skin-organotypic cocultures and the growth and branching morphogenesis of lung and kidney epithelial cells [46], [47]. Our results suggest that catS may interfere with BM network by cleaving the sites on nid-1 by which it binds to BM partners.

Other proteases, including serine proteases such as leukocyte elastase and MMPs, can also cleave nid-1 [12], [48], [49]. They mainly cleave nid-1 at two sites, one is within the flexible linker between the G1 and G2 domains and the other in the G3 globular domain, to generate stable fragments of 110 kDa and 100 kDa that have different laminin-binding activities [48]. Though free nid-1 is highly susceptible to proteolysis, it seems to be less sensitive when it is complexed with other BM [11], [48], [50]. However, our data shown that catS still rapidly hydrolyzes nid-1, even when it is complexed with laminins, type IV collagen or perlecan. Furthermore, catS efficiently and specifically cleaved nid-1 from a murine tumor-derived BM extract or from normal human placenta (data not shown), at both acidic and neutral pH, while its degradation is hampered in spleen lysates from *ctss* ^−/−^ mice, pretreated with inhibitors of other classes of proteases. Rheological measurements show that disrupting the network that nid-1 forms with other BM components by catS causes the mechanical features (elasticity and viscosity) of a reconstituted BM matrix to collapse. Our studies show for the first time that cysteine cathepsins, particularly catS, secreted by human keratinocytes participate with other identified classes of proteases in the proteolysis of nid-1 extracted from the BM. Fleischmajer et al. proposed that keratinocytes regulate the accumulation of nid-1 at the DEJ zone by inhibiting nid-1 synthesis or by activating proteolytic enzymes to degrade it [51]. As catS is regarded as a potent matrix remodeling protease, the degradation of nidogen is likely. However, the physiological relevance of nidogen hydrolysis by catS has never been explored and should be subject of further investigations. Nonetheless, loss of laminin, nidogens and structural disorganization observed in Sjögren’s syndrome (SS) [52] may in part attributable to cysteine cathepsins, and particularly to catS, which expression is up-regulated and activity is increased in the tears of NOD mice, a model of SS [53].

## Supporting Information

Figure S1 **Selectivity of human cathepsins B, K, L and S antibodies.** (A) Immunoblots of purified human cathepsins B, K, L and S. (B) Human keratins from epidermis (proteins of 50–70 kDa) were transferred to nitrocellulose membrane and immunodetected by polyclonal anti-keratin. No cross reactivity was observed with anti-cathepsins B, K, L and S antibodies.
(TIF)

Figure S2 **Comparative efficiencies of cathepsins B, S, K and L to degrade major BM constituents.** (A) Type IV-collagen (12 µg) was incubated in the absence or in the presence of cathepsins B, S, K and L (50–100 nM) in buffer A during 4 h. Similar assays were performed with laminin-211/221 (3.5 µg), perlecan (3 µg) and fibronectin (5 µg) As a control, each cathepsin (100 nM) was pre-incubated 30 min with the cysteine protease specific inhibitor E-64 (100 µM). Samples were loaded and separated by SDS-PAGE (10%) under reducing conditions. Gels were stained with Coomassie Blue. Percentages of residual BM proteins in the presence of cathepsins are shown +/− S.E.D. (B) BM matrix from EHS mouse sarcoma (ECM gel, 8 mg/ml) was incubated with cathepsins B, S, K and L (10–200 nM) at pH 5.5 for 4 h at 37°C. For controls, each cathepsin (200 nM) was incubated with E-64 (1 µM) before adding it to the BM extract.
(TIF)
