# Supplementary figures and images for: Cleavage of Nidogen-1 by Cathepsin S Impairs Its Binding to Basement Membrane Partners
Source: PLoS One. 2012 Aug 28;7(8):e43494. doi: 10.1371/journal.pone.0043494 (PMC3429489; doi:10.1371/journal.pone.0043494)

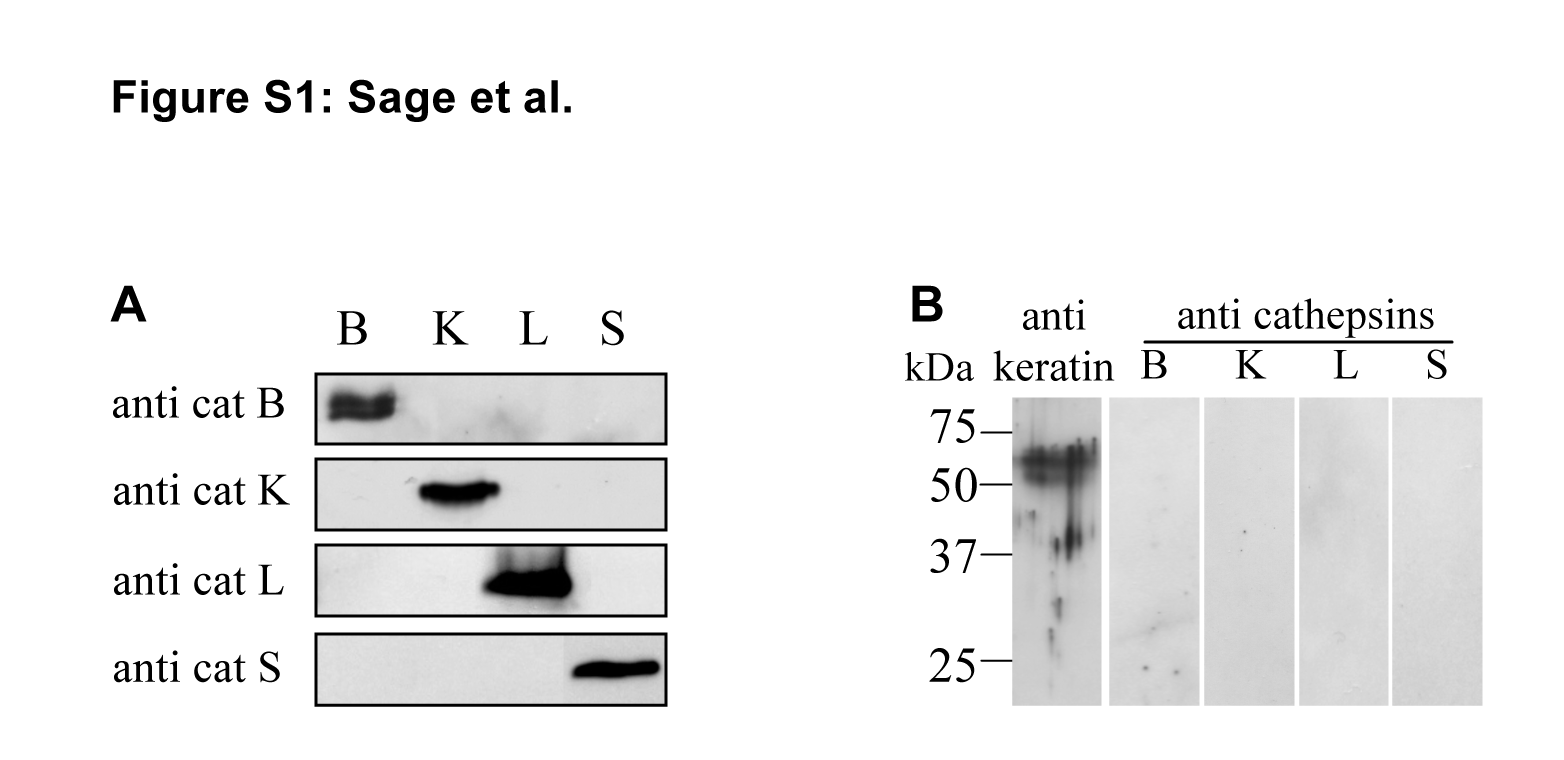

Supplement: Figure S1 — Selectivity of human cathepsins B, K, L and S antibodies. (A) Immunoblots of purified human cathepsins B, K, L and S. (B) Human keratins from epidermis (proteins of 50–70 kDa) were transferred to nitrocellulose membrane and immunodetected by polyclonal anti-keratin. No cross reactivity was observed with anti-cathepsins B, K, L and S antibodies. (TIF) [file pone.0043494.s001.tif]

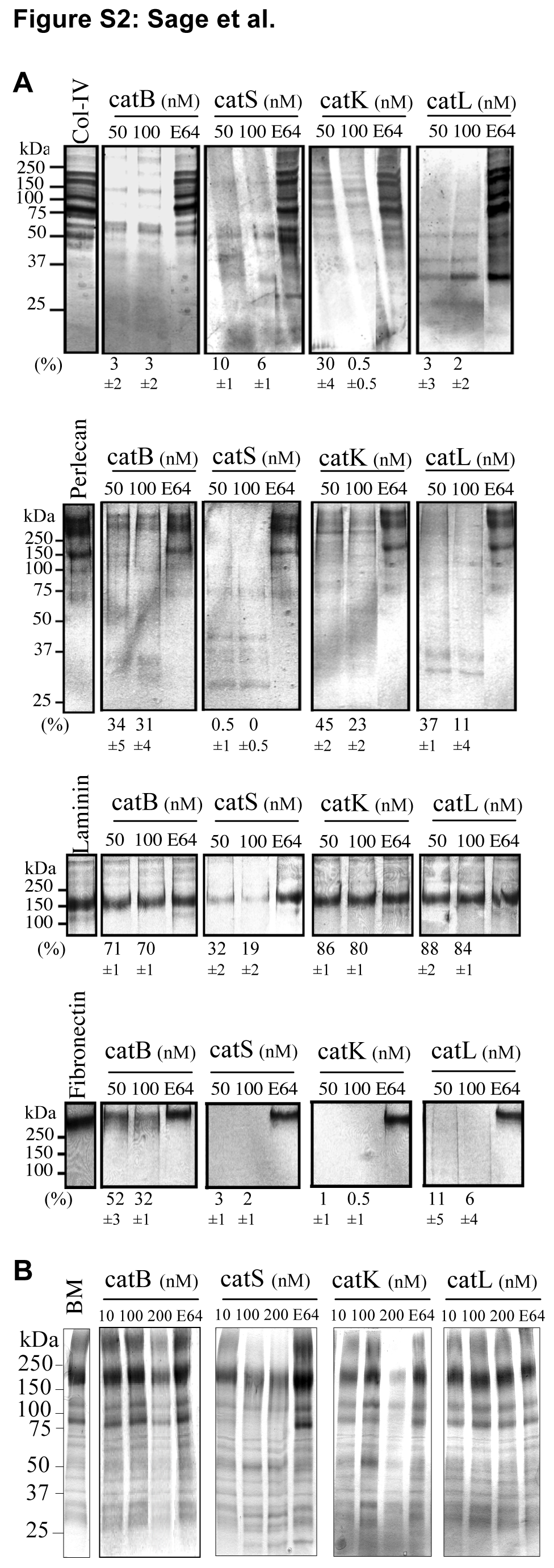

Supplement: Figure S2 — Comparative efficiencies of cathepsins B, S, K and L to degrade major BM constituents. (A) Type IV-collagen (12 µg) was incubated in the absence or in the presence of cathepsins B, S, K and L (50–100 nM) in buffer A during 4 h. Similar assays were performed with laminin-211/221 (3.5 µg), perlecan (3 µg) and fibronectin (5 µg) As a control, each cathepsin (100 nM) was pre-incubated 30 min with the cysteine protease specific inhibitor E-64 (100 µM). Samples were loaded and separated by SDS-PAGE (10%) under reducing conditions. Gels were stained with Coomassie Blue. Percentages of residual BM proteins in the presence of cathepsins are shown +/− S.E.D. (B) BM matrix from EHS mouse sarcoma (ECM gel, 8 mg/ml) was incubated with cathepsins B, S, K and L (10–200 nM) at pH 5.5 for 4 h at 37°C. For controls, each cathepsin (200 nM) was incubated with E-64 (1 µM) before adding it to the BM extract. (TIF) [file pone.0043494.s002.tif]
